# Using concept mapping to prioritize barriers to diabetes care and self-management for those who experience homelessness

**DOI:** 10.1186/s12939-021-01494-3

**Published:** 2021-07-09

**Authors:** Eshleen K. Grewal, Rachel B. Campbell, Gillian L. Booth, Kerry A. McBrien, Stephen W. Hwang, Patricia O’Campo, David J. T. Campbell

**Affiliations:** 1 Department of Medicine, Cumming School of Medicine, University of Calgary, Calgary, Canada; 2 Dalla Lana School of Public Health, University of Toronto, Toronto, Canada; 3 Department of Medicine, Faculty of Medicine, University of Toronto, Toronto, Canada; 4 MAP Centre for Urban Health Solutions, St. Michael’s Hospital, Unity Health Toronto, Toronto, Canada; 5 Institute for Clinical Evaluative Sciences, Toronto, Canada; 6 Department of Family Medicine, Cumming School of Medicine, University of Calgary, Calgary, Canada; 7 Department of Community Health Sciences, Cumming School of Medicine, University of Calgary, Calgary, Canada; 8 Department of Cardiac Sciences, Cumming School of Medicine, University of Calgary, Calgary, Canada

**Keywords:** Diabetes mellitus, Homeless persons, Patient engagement research, Patient priorities, Community-based participatory research

## Abstract

**Background:**

Diabetes is a chronic medical condition which demands that patients engage in self-management to achieve optimal glycemic control and avoid severe complications. Individuals who have diabetes and are experiencing homelessness are more likely to have chronic hyperglycemia and adverse outcomes. Our objective was to collaborate with individuals experiencing homelessness and care providers to understand the barriers they face in managing diabetes, as a first step in identifying solutions for enhancing diabetes management in this population.

**Methods:**

We recruited individuals with lived experience of homelessness and diabetes (i.e. clients; *n* = 32) from Toronto and health and social care providers working in the areas of diabetes and/or homelessness (i.e. providers; *n* = 96) from across Canada. We used concept mapping, a participatory research method, to engage participants in brainstorming barriers to diabetes management, which were subsequently categorized into clusters, using the Concept Systems Global MAX software, and rated based on their perceived impact on diabetes management. The ratings were standardized for each participant group, and the average cluster ratings for the clients and providers were compared using t-tests.

**Results:**

The brainstorming identified 43 unique barriers to diabetes management. The clients’ map featured 9 clusters of barriers: *Challenges to getting healthy food*, *Inadequate income*, *Navigating services, Not having a place of your own*, *Relationships with professionals*, *Diabetes education*, *Emotional wellbeing*, *Competing priorities*, and *Weather-related issues*. The providers’ map had 7 clusters: *Access to healthy food*, *Dietary choices in the context of homelessness*, *Limited finances, Lack of stable, private housing*, *Navigating the health and social sectors*, *Emotional distress and competing priorities*, and *Mental health and addictions*. The highest-rated clusters were *Challenges to getting healthy food* (clients) and *Mental health and addictions* (providers). *Challenges to getting healthy food* was rated significantly higher by clients (*p* = 0.01) and *Competing priorities* was rated significantly higher by providers (*p* = 0.03).

**Conclusions:**

Experiencing homelessness poses numerous barriers to managing diabetes, the greatest of which according to clients, is challenges to getting healthy food. This study showed that the way clients and providers perceive these barriers differs considerably, which highlights the importance of including clients’ insights when assessing needs and designing effective solutions.

**Supplementary Information:**

The online version contains supplementary material available at 10.1186/s12939-021-01494-3.

## Background

Diabetes mellitus is a commonly occurring chronic medical condition that is associated with a high burden of mortality and morbidity. In 2018, 34.2 million Americans, or 10.5 % of the population, had diabetes and approximately 1.5 million Americans were newly diagnosed that year [1]. The risk of mortality for people with diabetes is two times greater than it is for people without diabetes, and having diabetes can reduce life expectancy by 5–15 years [2]. Chronically high blood glucose levels can result in an increased risk of diabetes-related complications over time, which include: nerve damage, kidney disease, blindness, and vascular disease [3]. To avoid adverse outcomes, people with diabetes must make decisions about their management frequently and on an ongoing basis. There is evidence to suggest that the combination of self-management education and self-management support can result in improvements in glycemic control through improved self-care behaviours, which in turn can reduce the risk of developing complications [4]. Participation in diabetes education, therefore, is critical for improving knowledge and self-efficacy, which enables patients to engage in healthy behaviours [4]. These healthy behaviours, including smoking cessation, dietary modification, and regular physical activity, in combination with pharmacotherapy, can reduce the risk for complications such as major adverse cardiac events [5]. Other behaviours such as self-monitoring of blood glucose levels can empower patients, improve treatment adherence, and assist diabetes care professionals in making decisions about treatment [6].

Among those with diabetes, people with lived experience of homelessness (PWLEH) are more likely than the general population to have elevated blood glucose levels [7]. Homelessness is defined by a lack of stable and safe housing, but it is often accompanied by inadequate income, mental and physical health problems, difficulty accessing health care and social supports, substance use disorders, previous involvement with the justice system, and adverse childhood experiences [8, 9]. Although health issues may lead to homelessness, homelessness also greatly affects health, making it challenging for people who are experiencing homelessness to access care [8, 10] and engage in self-management, especially when they have a chronic medical condition like diabetes, for which management is complex.

There are many barriers that can make it challenging to manage diabetes for PWLEH. Accessing health care services can involve long wait times and an appreciable amount of time may be spent travelling to and from appointments [11]. In addition to time, there is a financial burden associated with managing diabetes, as the costs of medications and blood glucose testing supplies may not be covered by public health insurance programs, even within Canada’s universal health system [7, 11, 12]. Barriers specific to homelessness that have been identified in the literature include concerns about food insecurity related to low-quality foods available in shelters, as well as challenges with safely storing medications and supplies, and strict shelter schedules with respect to eating meals and taking medications [7, 13]. Travelling to appointments often involves the use of public transportation with associated costs. As many PWLEH do not have a fixed mailing address or reliable access to a phone, issues contacting patients are also common [8, 14], making it difficult to schedule and notify patients of appointments [15]. Perceived feelings of being unwelcome in clinical settings can also prevent people who are experiencing homelessness from seeking health care services [16]. Other priorities, namely, obtaining the necessities of life (food, shelter, etc…), drug and alcohol use, and mental health or cognitive issues can also interfere with diabetes self-management [7, 13, 17].

While there are many barriers and challenges to diabetes management, the importance of these in relation to one another is not well known, nor is it well known if these priorities differ between providers and PWLEH. Other studies that have compared the perspectives of service users with those of service providers have found differences of opinions between the two groups [18, 19]. One such study asking community members and service providers about which social and mental health services should be made available at a new medical clinic in the community found that community members and service providers had different views regarding which services were most needed [19]. Another study aimed to identify existing gaps in services for youth, where the opinions of the youth differed from those of the service providers regarding importance, but not in terms of ‘what to do first’ [18]. Traditionally, homeless-serving agencies have provided services based on their own beliefs about their clients’ needs instead of directly asking their clients what they need [20]. For instance, they may have focused on providing mental health and substance use services because the rates of mental illness and substance use are high among this population, or they may have prioritized housing because this population is characterized by a lack of a stable home, but those services may not address the most pressing issues for people who are experiencing homelessness [20] and may result in service gaps and barriers. The perspectives of service users, therefore, can be useful for service providers because those insights can help the providers understand how to tailor their services to better fill the gaps in service provision and remove barriers to accessing services, based on their clients’ priorities.

Given the complexity of diabetes management, myriad barriers that can affect adherence, and the potential for differences in perspectives between providers and PWLEH, we sought to collaborate with people who had lived experience of homelessness and diabetes, as well as health and social care providers who work with people experiencing homelessness and/or people with diabetes, to prioritize the challenges faced by this population in managing diabetes. The objective of this study was to determine the perceived relative impact of barriers to diabetes management and whether the perceptions about the impact of those barriers differed between providers and PWLEH.

## Methods

### Study design

 We used a semi-quantitative, participatory methodology known as concept mapping. We used concept mapping because it can be used to create visual representations that depict the combined thoughts of a group, by integrating input from multiple people using quantitative data analysis techniques [21]. This methodology enabled us to gather insights from a group of PWLEH who have diabetes (herein referred to as the clients) and from various providers representing different areas of health and social care (herein referred to as the providers) and to quantitatively compare their collective feedback. Our study was approved by the research ethics boards of the University of Calgary and Unity Health Toronto/St. Michael’s Hospital.

### Study participants & recruitment

#### Clients

The clients for this study were all recruited in Toronto, Ontario – Canada’s largest city and the city with the largest population of people experiencing homelessness [22]. The prevalence of diabetes among PWLEH in Toronto is thought to be similar to the prevalence among the general population [23] and barriers to managing diabetes previously identified by PWLEH in Toronto [7] are similar to the barriers identified by PWLEH in other cities [24].

Eight of the clients in this study were recruited as part of a larger community-based participatory research project designed to understand what it is like to live with diabetes while experiencing homelessness or housing instability and to propose and develop potential interventions for this population [25]. These eight participants met regularly in Toronto for study-related activities. In addition, we recruited several other participants to take part in this concept mapping exercise, to increase the sample size. To be eligible to participate, the clients had to: be older than 18 years, have a history of diabetes with a duration greater than two years, and have experienced homelessness within the previous two years. Homelessness in this study was defined similarly to the definition offered by the Canadian Observatory on Homelessness; we included participants whose living situations could be described as unsheltered, emergency sheltered, or provisionally sheltered [26]. Colleagues and advocates in the homeless-serving sector were consulted to determine the best places for recruiting the target population. Posters were put up in shelters, at homeless-serving agencies, and health clinics catering to the homeless population for recruitment. Established programs serving people who were experiencing homelessness or housing instability also helped with recruitment by advertising an information session to their program attendees. We explained the study to interested parties and obtained informed consent.

#### Providers

The service providers who took part in this study had previously been recruited as participants in another study of diabetes care for PWLEH [27]. They comprised professionals from four main categories of care: diabetes care professionals who worked primarily with inner-city or homeless populations, other health care professionals who worked primarily with inner-city or homeless populations, endocrinologists or diabetes care providers who did not specifically focus on inner-city or homeless populations, and other stakeholders, including frontline staff in shelters and social care providers. The providers were recruited from five major Canadian cities (Toronto, Ottawa, Edmonton, Calgary, and Vancouver), and they participated in this study remotely. Although the barriers to diabetes self-management may be similar across cities for PWLEH, the services available in each city differ [27]. Services in the health sector are largely impacted by funding and approval from provincial governments, which may have different priorities, and services in the homeless-serving sector are often provided by non-profit organizations, which rely to a great extent on donor funding for service provision. For these reasons, services that exist in one city may not exist in another, and we felt that it would be valuable to gain input from providers across different jurisdictions about their perspectives.

### Data collection

**Fig. 1:**
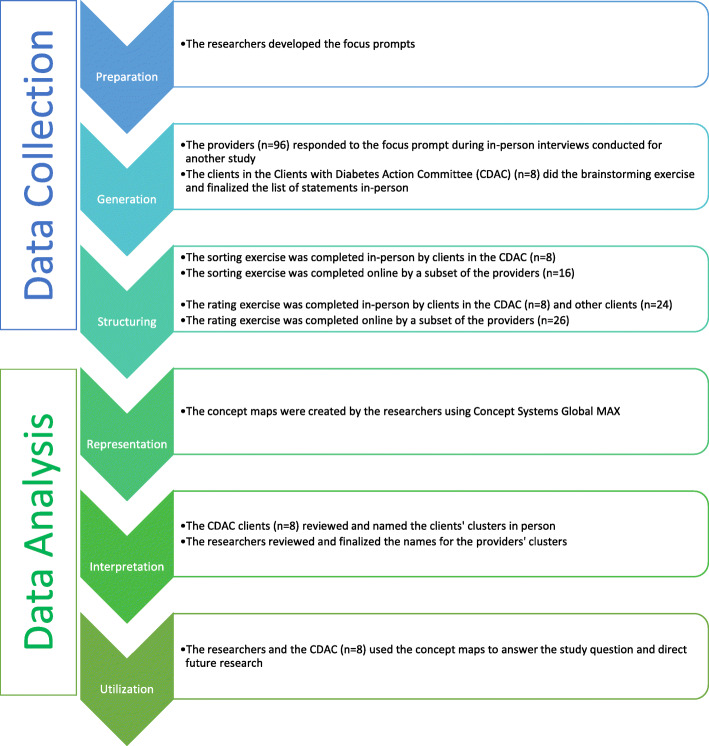
The concept mapping process

The concept mapping process involves six steps and is depicted in Fig. 1. The initial three steps relate to data collection: preparation, generation of statements, and structuring of statements [28].

In the first step, preparation, the researchers must determine what the focus of the study will be. Usually, this involves two separate focus statements. The first statement is referred to as the focus prompt or the brainstorming focus, and it is developed to guide the brainstorming exercise in the second step. The second focus statement, called the rating focus, defines the factor(s) upon which the brainstormed statements will be rated in the rating exercise of the third step [28, 29]. The focus prompt for the brainstorming exercise was, *‘What are some ways, good or bad, that diabetes might be affected by homelessness?’*. The rating prompt was: *‘What impact does [that factor] have on diabetes care and self-management (on a scale from no impact to high impact), specifically for people who are struggling with homelessness or housing instability?’*.

Generation involves a brainstorming exercise, in which the participants are provided with the focus prompt and asked to brainstorm statements in response [28]. The brainstorming exercise is done individually by each participant, and eventually, the ideas are combined and refined to form one set of statements [29]. Eight clients did the brainstorming exercise during an in-person group meeting. The providers had been individually interviewed by members of the research team, who then shared the providers’ ideas with the clients during their brainstorming session. The researchers recorded the ideas from the clients and the providers and refined the list of statements with the clients. The providers did not take part in the refinement of statements, as this aspect of the brainstorming exercise was done in person during a meeting with the clients.

Structuring involved two different exercises, sorting and rating. For the sorting exercise, each participant was asked to group individual statements into categories based on similarity, in a way that was meaningful to that individual [21, 30]. The statements could not all be sorted into one pile, nor could each statement be in its own pile [29]. “Miscellaneous” piles with a group of statements that did not have a specific theme or link to each other were also discouraged. These instructions were explained to participants at the outset of the exercise and facilitators were present to help clarify instructions. The clients completed this exercise manually with statements printed on individual cue cards, while the providers completed this exercise using a tailor-made online platform. For the rating exercise, a Likert scale ranging from zero to four was used to rate each statement. A rating of zero meant the statement had no impact on diabetes control and self-management, and a rating of four meant it had an extreme impact on diabetes control and self-management. This exercise was done on paper forms by the clients and online by the providers.

### Data analysis

The final three steps of the concept mapping process relate to data analysis: representation of statements, interpretation of maps, and utilization of maps [28].

In the representation step, sorting and rating data were analyzed using concept mapping software (Concept Systems Global Max, 2020, Ithaca NY) [30, 31]. The representation step was done separately for the clients and the providers: the clients’ data were analyzed to create a clients’ cluster map and the providers’ data were analyzed to produce a providers’ cluster map. By having two cluster maps, rather than one, we were able to see how the number of clusters, the names of clusters, and the sorting of the statements, differed between the two participant groups.

The sorting data were analyzed using multidimensional scaling analysis, a technique that plots each statement as a point on a map. Statements that are closer together on the map were grouped together during the sorting exercise more often than statements that are farther apart. Multidimensional scaling was used to generate a matrix for each participant with one row and one column representing each of the statements [29]. In this study, the matrix had 43 rows and 43 columns. Each cell in the matrix corresponds to two statements, the row statement and the column statement. If those two statements were sorted into the same pile by the participant, the cell is given a value of one, and if they were not sorted into the same pile, the cell is assigned a value of zero [21, 29]. This is done with each participant’s data so that there are as many separate matrices as there are participants in the study. All of the individual matrices are then summed to create a similarity matrix indicating how many participants sorted each pair of statements into the same pile [29]. This similarity data is used to create a two-dimensional point map, where the distance between two points on the map corresponds to their similarity [21].

These points were then grouped together to form clusters through hierarchical cluster analysis. Hierarchical cluster analysis uses the data from the point map to separate the points into non-overlapping clusters, developing a cluster map. The number of clusters in the final cluster map is determined by the participants and the researchers so that the map is presented in a way that is meaningful and useful to them [21]. This analysis begins by considering each statement to be in a cluster of its own, and it combines two clusters at a time, reducing the number of overall clusters until all the statements are in the same cluster. The statements are combined based on proximity, meaning that the two points that are closest together on the point map would be the first two points to form a cluster together [29]. The reliability, or goodness-of-fit of the map, is determined by calculating the final stress of the model [32]. Lower stress values suggest a better fit. Stress values in concept mapping data tend to be higher than in other multidimensional scaling analyses [33], therefore there is no accepted standard, however, it has been reported that an acceptable value for studies of this nature is less than 0.30 [34].

An average rating is then calculated for each cluster using the ratings for each of the individual statements in that cluster. Upon reviewing the average cluster ratings, it was clear that the clients and providers had rated the statements very differently, such that the clients had generally given lower ratings whereas the providers had given higher ratings, which resulted in lower average cluster ratings for the clients’ clusters and higher average cluster ratings for the providers’ clusters. To determine whether there was in fact a difference in the ratings between the two groups, the ratings were standardized and compared using unpaired two-sided t-tests, with an alpha of < 0.05. To standardize, the participants’ ratings for each statement were subtracted from the average rating of all the statements for their participant group (i.e., the clients’ average rating or the providers’ average rating). Both groups had different numbers of clusters with different numbers of statements in them, which would have been difficult to compare, so it was necessary to use the same cluster structure for both groups. We chose to compare based on the clients’ cluster structure because we wanted the analysis to be client-centred and because the clients had reviewed the clusters and the statements that were in each cluster, so the final structure reflected their feedback. We used unpaired t-tests to quantitatively compare the standardized cluster ratings to determine whether there was a statistically significant difference between the ratings of the clients and those of the providers for each cluster in the patient cluster map.

In the interpretation step, the maps and reports generated in the previous step are used to help frame the interpretation of the data. We assessed point maps, cluster maps, cluster reports with statements, and raw ratings reports. In this step, some of the clients were reconvened for an in-person meeting to discuss any changes that needed to be made to the maps and to provide input on the names of each of the clusters. The providers were not able to come together for a meeting, so their clusters were named by the researchers.

In the final step, utilization, the concept maps are reviewed to determine how they can be utilized to answer the study question [30]. In this study, the findings from the clients’ concept mapping research informed the subsequent research program in providing the topics of interest for the participatory photovoice project that ensued [35].

## Results

There were 32 clients and 96 providers who took part in this study: Although there were 128 participants altogether, only a subset of those participants took part in each stage of the concept mapping process (Fig. 1). For instance, only eight of the 32 clients took part in the brainstorming and sorting exercises, but all 32 took part in the rating exercise. For the providers, all 96 took part in the brainstorming exercise, but only 16 and 26 of these individuals took part in the sorting and rating exercises, respectively. The final sample size is consistent with what has typically been reported in other concept mapping projects [34].

### Characteristics of the clients

The demographic characteristics of the clients are presented in Table 1. All of the clients were over the age of 25 and just over half identified as men. None of the clients were sleeping rough at the time of this study, while a quarter of the clients were in private housing with the remainder in shelter or tenuous, transitional, or community housing. Collectively, they had experienced homelessness or housing instability for a median length of two years and had been living with diabetes for a median length of seven years. A majority of the clients had experienced some diabetes-related complications and had physical or mental health comorbidities as well. The most commonly reported method of managing diabetes was the use of oral medications, which were utilized by three-quarters of the clients, although approximately one-third of the clients used injectable agents (including insulin). The clients also saw a variety of care providers for the treatment of their diabetes; about three-quarters saw a family physician, one-fifth saw a pharmacist, and one-third saw a medical specialist, a diabetes nurse, and a diabetes dietitian, respectively.

**Table 1:** Demographic characteristics of the clients (*n* = 32)

Characteristic	Number (%)
**Age**	
< 45	5 (15.6)
45–54	5 (15.6)
55–64	12 (37.5)
65+	9 (28.1)
Did not respond	1 (3.1)
**Gender**	
Woman	13 (40.6)
Man	18 (56.3)
Did not respond	1 (3.1)
**Ethnicity**	
White/Caucasian	13 (40.6)
Other	18 (56.3)
Did not respond	1 (3.1)
**Housing status**^a^	
Rough sleeping	0 (0)
Stable resident of shelter	14 (43.8)
Tenuous/Transitional housing	4 (12.5)
Community housing	5 (15.6)
Private residence	8 (25)
Did not respond	2 (6.3)
**Length of time experiencing housing instability**	
Years	Median = 2.0, IQR = 3.0
Did not respond	7
**Type of diabetes**	
Type 2	28 (87.5)
Type 1 or Other	3 (9.4)
Did not respond	1 (3.1)
**Length of time living with diabetes**	
Years	Median = 7.0, IQR = 11.5
Did not respond	3
**Treatment of diabetes**	
Lifestyle (diet and exercise)	18 (56.3)
Oral medications	25 (78.1)
Injectable medications (including insulin)	10 (31.3)
Did not respond	1 (3.1)
**Diabetes care providers**	
Family doctor	23 (71.9)
Specialist doctor (internal medicine, endocrinologist)	10 (31.3)
Diabetes nurse	11 (34.4)
Diabetes dietician	10 (31.3)
Pharmacist	6 (18.8)
Other	8 (25)
Did not respond	1 (3.1)
**Diabetes complications**	
Heart disease/heart attacks/strokes	10 (31.3)
Foot ulcers (wounds), gangrene, amputations	3 (9.4)
Kidney problems (nephropathy)	6 (18.8)
Diabetes eye problems (retinopathy)	9 (28.1)
Burning, tingling, numbness in toes and feet	16 (50)
Did not respond	8 (25)
**Comorbidities**	
High blood pressure	19 (59.4)
High cholesterol	16 (50)
Obesity	16 (50)
Sleep apnea	14 (43.8)
Depression	16 (50)
Anxiety problems (panic attacks, general anxiety, phobias)	16 (50)
Psychosis (schizophrenia, schizoaffective, delusional disorder)	6 (18.8)
Alcohol addiction	8 (25)
Drug addiction	8 (25)
Did not respond	4 (12.5)

^a^one participant chose 2 housing options, therefore n sums to 33

### Characteristics of the providers

Only eight of the providers completed the demographic survey. Most (6/8) were women, half were between the ages of 55 and 64, and 5/8 had 20 or more years of experience working with patients that have diabetes and/or complex social needs, while the rest had 15–19 years of experience. The providers also had a variety of different qualifications and titles including, registered dietitian, registered nurse, certified diabetes educator, family physician, program administrator/manager, and executive director. They also worked in a variety of settings such as community/private family medicine practices, specialty practices, community health centres, community pharmacies, academic/public family medicine practices, and homeless shelters.

### Concept mapping

During the brainstorming exercise, the participants generated a list of statements representing barriers to self-management, starting with a large list, which was then refined by removing duplicates and combining similar statements. The resulting, final version of the list contained 43 statements (Table 2).

**Table 2:** List of unranked and unsorted statements from the brainstorming exercise

1	Food that is provided in shelters and community meals is not diabetic friendly
2	Unhealthy “comfort food” is a source of joy in an otherwise difficult day
3	Diabetic appropriate foods are unaffordable
4	Portion control and making healthy choices is hard when you don’t know where the next meal is coming from
5	Not having a kitchen where one can prepare healthy food
6	Getting out of the weather or accessing Wi-Fi requires purchase of fast food
7	Food available at food banks is not diabetic appropriate
8	Meals or food is only provided at set times in shelter
9	It is difficult to navigate the network of diabetes care providers (to get eye exams, blood work, urine tests, foot exams/care, etc.)
10	Past experiences with discrimination, racism, and/or prejudice in health care settings makes engaging in care undesirable
11	Not having trusting relationships with healthcare providers
12	It is difficult to keep track of days for attending appointments
13	Not having a way for doctors’ offices and diabetes care providers to get in touch (i.e. phone, consistent address, etc.)
14	Not having an affordable and convenient way to get to appointments
15	It is difficult to access health services due to lack of health insurance card or ID
16	Community and government social support programs are hard to navigate
17	Social assistance levels are insufficient
18	Not having enough knowledge about diabetes and its treatment
19	Mainstream diabetes education programs are not relevant to life circumstances
20	Mainstream diabetes education programs are not offered at a convenient place or time
21	Not having diabetes appropriate footwear
22	The danger of exposure to fingers and toes when sleeping outside
23	Not having reliable access to a bath or shower for foot care
24	Not having a secure place to store medications (where they won’t get stolen)
25	Not being able to afford medications
26	Having staff administer medications to patient
27	Keeping track of time of day for taking medications
28	Managing the interaction between recreational substances (alcohol/drugs) and diabetes treatment is difficult
29	The fear of having a low blood sugar in shelter or alone
30	There are too many other health concerns to deal with
31	There are too many non-health-related concerns to deal with (i.e. housing, relationships, money, etc.)
32	Mental health challenges make it hard to focus on giving diabetes the attention it requires
33	Addictions make it hard to focus on giving diabetes the attention it requires
34	Not being able to afford organized physical activities
35	Local weather makes it difficult to be active outdoors year-round
36	Testing supplies/pen tips are unaffordable
37	Not having a place to store diabetes supplies
38	Lack of privacy on the street or in shelter
39	Stigma, intimidation, or violence from peer community in shelter or transitional housing
40	Lack of family or other close personal connections, or negative influence/impact
41	Shelter staff and case managers don’t understand diabetes
42	High stress levels due to housing situation/frequent moves
43	Housing struggles lead to hopelessness and lack of concern about diabetes

**Fig. 2:**
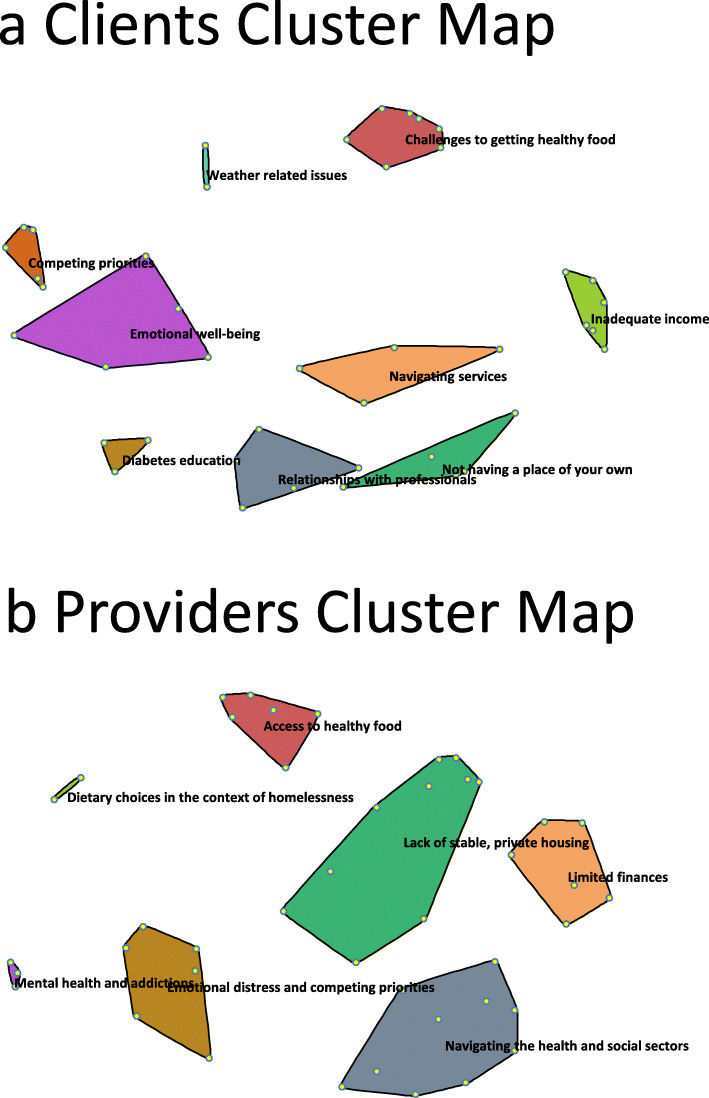
Cluster maps for the clients and the providers. **a**. Clients’ cluster map. **b**. Providers’ cluster map

Using the data from the sorting exercises, the statements were plotted in relation to one another and presented as point maps through multidimensional scaling, and the points were then grouped to form cluster maps (Fig. 2a and b) through hierarchical cluster analysis. The clients’ cluster map is based on sorting data from eight participants, and it consists of nine clusters, with a final stress value of 0.2987. Those clusters were named: (1) *Challenges to getting healthy food*, (2) *Inadequate income*, (3) *Navigating services*, (4) *Not having a place of your own*, (5) *Relationships with professionals*, (6) *Diabetes education*, (7) *Emotional well-being*, (8) *Competing priorities*, and (9) *Weather-related issues*. The providers’ cluster map had seven clusters, made using sorting data from 16 participants, and had a final stress value of 0.2521. The clusters were named: (1) *Access to healthy food*, (2) *Dietary choices in the context of homelessness*, (3) *Limited finances*, (4) *Lack of stable, private housing*, (5) *Navigating the health and social sectors*, (6) *Emotional distress and competing priorities*, and (7) *Mental health and addictions*. Appendix A (see Additional File 1) contains tables that list which statements were in each cluster for both the clients and the providers.

The rating exercise was completed by 32 clients and 26 providers. The ratings for the individual statements were used to calculate an average cluster rating. The clients’ clusters, arranged from the cluster with the highest average rating to the cluster with the lowest average rating, with the average rating in parentheses (out of a total of four), are: (1) *Challenges to getting healthy food* (2.08), (2) *Weather-related issues* (1.83), (3) *Emotional well-being* (1.65), (4) *Inadequate income* (1.65), (5) *Competing priorities* (1.60), (6) *Relationships with professionals* (1.43), (7) *Diabetes education* (1.35), (8) *Not having a place of your own* (1.23), and (9) *Navigating services* (1.18). Providers’ clusters, by contrast, were: (1) *Mental health and addictions* (3.54), (2) *Emotional distress and competing priorities* (3.42), (3) *Dietary choices in the context of homelessness* (3.12), (4) *Navigating the health and social sectors* (3.00), (5) *Limited finances* (2.97), (6) *Lack of stable, private housing* (2.88), and (7) *Access to healthy food* (2.79).

**Fig. 3:**
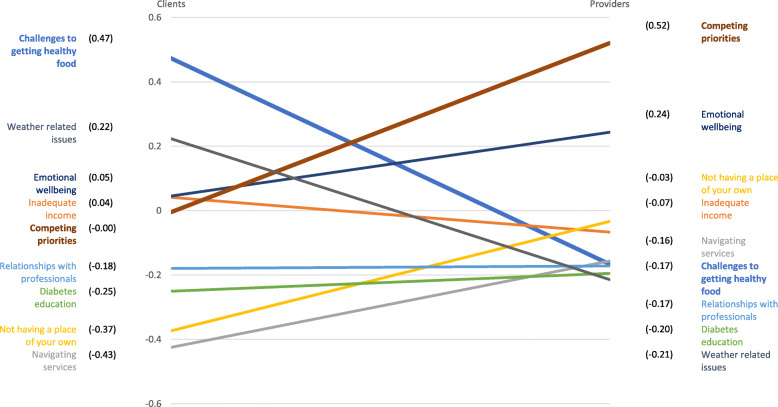
Graphical representation of the average standardized cluster ratings

Using the grouping of clusters defined by clients, we calculated standardized cluster averages for the two groups (Fig. 3). There were two clusters for which the standardized averages differed in a statistically significant manner, *Challenges to getting healthy food* (standardized difference = 0.6396; *p* = 0.013) and *Competing priorities* (standardized difference=-0.5250; *p* = 0.026). The clients had a higher rating for *Challenges to getting healthy food* than the providers and the providers had a higher rating for *Competing priorities*. While trends were suggesting potential differences in the other clusters, these were the only two that reached statistical significance, due to the relatively small sample size for this quantitative analysis.

## Discussion

The clients and providers identified a plethora of barriers to diabetes management, ultimately resulting in a refined list of 43 unique barriers. The clients’ and providers’ sorting data resulted in concept maps with distinct cluster names and configurations representing differing perceptions of barriers to managing diabetes while experiencing homelessness. Both the clients’ and the providers’ clusters represented themes related to access to healthy food; financial limitations; housing; health and social care; and psychosocial wellbeing. The clients chose to have a cluster titled *Relationships with professionals*, whereas the providers’ map included those barriers in the *Navigating the health and social sectors* and *Lack of stable, private housing* clusters. While the clients’ map has two clusters representing similar themes: *Navigating services* and *Not having a place of your own*, the clients saw the barriers related to relationships as being distinct from those related to navigation issues or housing issues and chose to have a separate cluster for them. These findings confirm the notion that the perspectives of clients/patients/service users and service providers regarding barriers to diabetes management may be different, and that the reasoning behind patients’ differing perspectives may not be evident to the providers. Traditionally, the identification of barriers and the creation of solutions has not meaningfully considered input from the individuals who have the most at stake, the patients/clients. Our findings highlight the importance of considering the patient perspective when designing solutions for enhancing diabetes management to address the barriers which have the greatest impact, according to patients.

Comparisons of the cluster ratings indicated that there were significant differences between the clients and the providers in the perceived impact of the barriers faced in diabetes management. The cluster related to challenges to accessing healthy food was the most influential from the client perspective, and it was rated significantly higher by the clients than the providers. The cluster representing priorities that compete with diabetes management was thought to be the most impactful by providers, and the rating for this cluster was significantly higher for the providers compared to the clients.

There are many reasons why accessing healthy food can be challenging for PWLEH. Many may rely on shelters or community kitchens for food so they must eat whatever is available, even if they have been advised to avoid such food by their healthcare providers [36]. The meals in shelters often have high amounts of sugar, starch and fat, and there are few fruits and vegetables available, which results in diets that are likely to be inappropriate for people with diabetes [7]. These shelters and community kitchens, however, also have limited resources and must often rely on food that is donated to them [24]. Sometimes, PWLEH may be unable to get three meals in a day so they are often eating when they can and hoarding extra food, so they have something to eat later. Alternatively, if they had access to affordable, nutritious food, they would not be worried about where their next meal is going to come from [37] and they would have greater diabetes self-management self-efficacy [38]. The lack of access to food is especially concerning for people who are using insulin, as they may use less than the prescribed amount of insulin for fear of developing hypoglycemia if they are not able to predict mealtimes reliably [15]. It is likely that because PWLEH need to negotiate their dietary intake daily, this rose to the top of the priority list for them. Providers may benefit from knowing that for PWLEH this is the most troubling aspect of diabetes self-management.

With regards to competing priorities, studies have noted that PWLEH tend to have many demands and prioritize things such as food, shelter, and employment above diabetes care and self-management practices [13]. When they continually face difficulties in meeting these basic needs, people may forego preventative care or sacrifice self-care [39]. This may partially explain why the providers believed that competing priorities have a great effect on diabetes management. As for why this cluster of factors was rated lower by clients, we hypothesize that this discrepancy may relate to lower health-related self-awareness in this population [40], or because clients did not fully grasp how their other issues may affect their diabetes, likely due to the fact that their social networks are often comprised of others who face similar issues. This is consistent with literature documenting that non-clinically trained people are typically less aware of the impact of the social determinants of health [41]. By contrast, healthcare providers are more likely to be informed about the social determinants of health and how diabetes care is affected significantly by the complete picture of patients’ lives [42]. It is also possible that barriers related to competing priorities were deemed less impactful by the clients because their greatest competing priority is accessing food, which had a separate cluster of its own, and had the highest average rating for the clients. The clients may also have felt that accessing healthy food is more important than other aspects of self-management if the diabetes education they received emphasized the importance of diet above all else.

The second highest-rated cluster for the clients was focused on issues related to the weather. Cold-related injuries are very common amongst PWLEH in Canada and they often result in emergency department visits [43]. One of the many complications of diabetes is reduced circulation, especially in the feet, so in cold weather foot care becomes even more important, as the winter weather can increase the risk for infections, frostbite and amputations [44, 45].

The providers’ third highest-rated cluster focused on a lack of stable, secure housing and while the clients had a similar cluster, for them, it received the second-lowest rating. Understandably, providers would consider this an important barrier, given that there is much in the literature describing the need to treat homelessness as a health issue [46] and suggesting that it is important for providers to help address housing concerns [15]. It is surprising, however, that this cluster was given such a low rating relative to the other clusters by the clients because participants in other qualitative studies have described housing as a foundational need that affects diabetes self-management in numerous ways [36, 47]. In one study, participants reported that being unstably housed is emotionally and physically draining, which makes it difficult to prioritize diabetes, and when the need for shelter is not met, there is no foundation from which they can pursue their health goals [47]. This view is in accordance with Maslow’s hierarchy of needs, which places basic needs such as food and shelter ahead of health [8, 15, 48]. Not having housing also means not having a secure place to store diabetes testing supplies such as glucometers or medications and insulin, and there is a fear that they may be stolen in a communal living arrangement such as an emergency shelter [36]. Furthermore, stable housing can provide a sense of consistency and control that can help with the routinization of diabetes care and allow some control over diet, while high housing costs can compete with the cost of diabetes care [47].

One of the strengths of this study is its participatory nature. We gathered input from PWLEH as well as a variety of providers who work in health and social settings, which ensured that we had diverse perspectives represented. The clients were able to review the concept maps and name the clusters, which meant that the final maps reflected their perspectives rather than the opinions of the researchers. This is important because the participants may see the value of having certain themes or representing the barriers a different way than the researchers. Concept mapping incorporates both qualitative and quantitative analyses in one process, which enables complex ideas to be explored in a short period of time, and the output of the quantitative analyses supplements and enhances the qualitative interpretations. Furthermore, the creation of visual representations through this combination of analyses provides structure and credibility to the results [30]. Additionally, it includes both individual and group activities, and the process in which these activities occur avoids some of the issues that are commonly experienced when using qualitative methodologies such as, the monopolization of group discussion time by one or two individuals, the likelihood of conformity bias, or the need for individuals to publicly discuss their personal opinions or experiences [30]. Another strength is that the participants were involved in the analysis of the data and they were able to interpret the concept maps that were created using their data. This methodology ensures that the thoughts of the participants are accurately reflected [28, 30]. The visual concept maps allowed us to display the associations between multiple themes and the rating data enabled comparison of the relative importance of each theme [28, 30].

There are also limitations to this study, one of which is that only eight clients completed the sorting exercise. This was due to the time commitment required of participants and the complexity of the task. Ideally, participants must take part in the brainstorming, sorting, and rating exercises, and then review the results and provide feedback, but it may be difficult to keep participants engaged throughout the whole process. However, despite this small sample, the resultant map still had an acceptable final stress value. Another limitation of this study is the lack of available demographic data about the providers, as only a small proportion of the providers completed the demographic survey. While it is unfortunate that we are unable to fully describe the group of providers who participated in this study, we do know that the roles they had and the settings they worked in varied considerably. Additionally, the providers did not have the opportunity to reflect on the concept map that was produced with their data. The research team decided to finalize the cluster map on their own because the providers were from five different cities, and it was not possible to plan a meeting for all of the providers.

## Conclusions

The participants in our study identified many of the same barriers that are described in other studies. However, this study is unique in that it allowed participants to rank these barriers from most to least impactful on diabetes management – and compared these rankings between PWLEH and providers. The results show that clients and providers differ in their understanding of barriers and the impact they have on diabetes management. Given that the clients in this study have indicated that difficulties with accessing healthy food are the greatest barriers to managing diabetes, future research and interventions aimed at improving diabetes management among PWLEH who have diabetes could focus on determining how to improve access to diabetes-appropriate food for this population.

## Supplementary Information

**Additional file 1.** Statements organized by cluster. Tables displaying the clients’ clusters with statements and the providers’ clusters with statements.

## Data Availability

The data pertaining to this study may be available upon request from the corresponding author.

## Abbreviation

PWLEH: people with lived experience of homelessness
