# Supplementary material for: Using concept mapping to prioritize barriers to diabetes care and self-management for those who experience homelessness
Source: Int J Equity Health. 2021 Jul 9;20:158. doi: 10.1186/s12939-021-01494-3 (PMC8272311; doi:10.1186/s12939-021-01494-3)
Supplement: Supplementary file 1 — Additional file 1. Statements organized by cluster. Tables displaying the clients’ clusters with statements and the providers’ clusters with statements. [file 12939_2021_1494_MOESM1_ESM.docx]

**Appendix A. Statements organized by cluster.**

Table A1. Clients’ clusters with statements

| Cluster |  | Statement |
| --- | --- | --- |
| Challenges to getting healthy food |  |  |
|  | 1 | Food that is provided in shelters and community meals is not diabetic friendly |
|  | 2 | Unhealthy "comfort food" is a source of joy in an otherwise difficult day |
|  | 3 | Diabetic appropriate foods are unaffordable |
|  | 4 | Portion control and making healthy choices is hard when you don't know where the next meal is coming from |
|  | 5 | Not having a kitchen where one can prepare healthy food |
|  | 7 | Food available at food banks is not diabetic appropriate |
|  | 8 | Meals or food is only provided at set times in shelter |
|  | 6 | Getting out of the weather or accessing WI-FI requires purchase of fast food |
| Inadequate income |  |  |
|  | 14 | Not having an affordable and convenient way to get to appointments |
|  | 17 | Social assistance levels are insufficient |
|  | 21 | Not having diabetes appropriate footwear |
|  | 25 | Not being able to afford medications |
|  | 34 | Not being able to afford organized physical activities |
|  | 36 | Testing supplies/pen tips are unaffordable |
| Navigating services |  |  |
|  | 9 | It is difficult to navigate the network of diabetes care providers (to get eye exams, blood work, urine tests, foot exams/care, etc.) |
|  | 13 | Not having a way for doctors’ offices and diabetes care providers to get in touch (i.e. phone, consistent address, etc.) |
|  | 16 | Community and government social support programs are hard to navigate |
|  | 15 | It is difficult to access health services due to lack of health insurance card or ID |
| Not having a place of your own |  |  |
|  | 23 | Not having reliable access to a bath or shower for foot care |
|  | 24 | Not having a secure place to store medications (where they won't get stolen) |
|  | 37 | Not having a place to store diabetes supplies |
|  | 38 | Lack of privacy on the street or in shelter |
|  | 29 | The fear of having a low blood sugar in shelter or alone |
| Relationships with professionals |  |  |
|  | 10 | Past experiences with discrimination, racism, and/or prejudice in health care settings makes engaging in care undesirable |
|  | 11 | Not having trusting relationships with healthcare providers |
|  | 26 | Having staff administer medications to patient |
|  | 39 | Stigma, intimidation, or violence from peer community in shelter or transitional housing |
|  | 41 | Shelter staff and case managers don't understand diabetes |
| Diabetes education |  |  |
|  | 18 | Not having enough knowledge about diabetes and its treatment |
|  | 19 | Mainstream diabetes education programs are not relevant to life circumstances |
|  | 20 | Mainstream diabetes education programs are not offered at a convenient place or time |
| Emotional well-being |  |  |
|  | 12 | It is difficult to keep track of days for attending appointments |
|  | 27 | Keeping track of time of day for taking medications |
|  | 40 | Lack of family or other close personal connections, or negative influence/impact |
|  | 42 | High stress levels due to housing situation/frequent moves |
|  | 43 | Housing struggles lead to hopelessness and lack of concern about diabetes |
| Competing priorities |  |  |
|  | 28 | Managing the interaction between recreational substances (alcohol/drugs) and diabetes treatment is difficult |
|  | 30 | There are too many other health concerns to deal with |
|  | 31 | There are too many non-health-related concerns to deal with (i.e. housing, relationships, money, etc.) |
|  | 32 | Mental health challenges make it hard to focus on giving diabetes the attention it requires |
|  | 33 | Addictions make it hard to focus on giving diabetes the attention it requires |
| Weather related issues |  |  |
|  | 22 | The danger of exposure to fingers and toes when sleeping outside |
|  | 35 | Local weather makes it difficult to be active outdoors year-round |

Table A2. Providers’ clusters with statements

| Cluster |  | Statement |
| --- | --- | --- |
| Access to healthy food |  |  |
|  | 1 | Food that is provided in shelters and community meals is not diabetic friendly |
|  | 3 | Diabetic appropriate foods are unaffordable |
|  | 5 | Not having a kitchen where one can prepare healthy food |
|  | 6 | Getting out of the weather or accessing WI-FI requires purchase of fast food |
|  | 7 | Food available at food banks is not diabetic appropriate |
|  | 8 | Meals or food is only provided at set times in shelter |
| Dietary choices in the context of homelessness |  |  |
|  | 2 | Unhealthy "comfort food" is a source of joy in an otherwise difficult day |
|  | 4 | Portion control and making healthy choices is hard when you don't know where the next meal is coming from |
| Limited finances |  |  |
|  | 14 | Not having an affordable and convenient way to get to appointments |
|  | 17 | Social assistance levels are insufficient |
|  | 21 | Not having diabetes appropriate footwear |
|  | 25 | Not being able to afford medications |
|  | 34 | Not being able to afford organized physical activities |
|  | 36 | Testing supplies/pen tips are unaffordable |
| Lack of stable, private housing |  |  |
|  | 22 | The danger of exposure to fingers and toes when sleeping outside |
|  | 23 | Not having reliable access to a bath or shower for foot care |
|  | 24 | Not having a secure place to store medications (where they won't get stolen) |
|  | 35 | Local weather makes it difficult to be active outdoors year-round |
|  | 37 | Not having a place to store diabetes supplies |
|  | 38 | Lack of privacy on the street or in shelter |
|  | 29 | The fear of having a low blood sugar in shelter or alone |
|  | 26 | Having staff administer medications to patient |
|  | 39 | Stigma, intimidation, or violence from peer community in shelter or transitional housing |
|  | 41 | Shelter staff and case managers don't understand diabetes |
| Navigating the health and social sectors |  |  |
|  | 9 | It is difficult to navigate the network of diabetes care providers (to get eye exams, blood work, urine tests, foot exams/care, etc.) |
|  | 10 | Past experiences with discrimination, racism, and/or prejudice in health care settings makes engaging in care undesirable |
|  | 11 | Not having trusting relationships with healthcare providers |
|  | 12 | It is difficult to keep track of days for attending appointments |
|  | 13 | Not having a way for doctors’ offices and diabetes care providers to get in touch (i.e. phone, consistent address, etc.) |
|  | 15 | It is difficult to access health services due to lack of health insurance card or ID |
|  | 16 | Community and government social support programs are hard to navigate |
|  | 18 | Not having enough knowledge about diabetes and its treatment |
|  | 19 | Mainstream diabetes education programs are not relevant to life circumstances |
|  | 20 | Mainstream diabetes education programs are not offered at a convenient place or time |
| Emotional distress and competing priorities |  |  |
|  | 27 | Keeping track of time of day for taking medications |
|  | 30 | There are too many other health concerns to deal with |
|  | 31 | There are too many non-health-related concerns to deal with (i.e. housing, relationships, money, etc.) |
|  | 40 | Lack of family or other close personal connections, or negative influence/impact |
|  | 42 | High stress levels due to housing situation/frequent moves |
|  | 43 | Housing struggles lead to hopelessness and lack of concern about diabetes |
| Mental health and addictions |  |  |
|  | 28 | Managing the interaction between recreational substances (alcohol/drugs) and diabetes treatment is difficult |
|  | 32 | Mental health challenges make it hard to focus on giving diabetes the attention it requires |
|  | 33 | Addictions make it hard to focus on giving diabetes the attention it requires |
